# Xiaoxuming Decoction: A Traditional Herbal Recipe for Stroke With Emerging Therapeutic Mechanisms

**DOI:** 10.3389/fphar.2021.802381

**Published:** 2021-12-14

**Authors:** Qian Zhang, Yue Wang, Aiwen Chen, Xinwei Huang, Qianyu Dong, Zhen Li, Xiaofei Gao, Tingmei Wu, Wanrong Li, Peilin Cong, Hanxi Wan, Danqing Dai, Mengfan He, Huazheng Liang, Shaoshi Wang, Lize Xiong

**Affiliations:** ^1^ Translational Research Institute of Brain and Brain-Like Intelligence, Shanghai Fourth People’s Hospital, School of Medicine, Tongji University, Shanghai, China; ^2^ Clinical Research Center for Anesthesiology and Perioperative Medicine, Tongji University, Shanghai, China; ^3^ Department of Neurology and Rehabilitation, Shanghai YangZhi Rehabilitation Hospital (Shanghai Sunshine Rehabilitation Center), School of Medicine, Tongji University, Shanghai, China; ^4^ Department of Anesthesiology, Shanghai Fourth People’s Hospital, School of Medicine, Tongji University, Shanghai, China

**Keywords:** xiaoxuming decoction, ischemic stroke, effective compound group, active compound, molecular mechanism, pericytes

## Abstract

Xiaoxuming decoction (XXMD) has been traditionally used to manage stroke though debates on its clinical efficacy were present in the history. Till nowadays, it is still one of the most commonly used herbal recipes for stroke. One of the reasons is that a decent proportion of ischemic stroke patients still have residue symptoms even after thrombolysis with rt-PA or endovascular thrombectomy. Numerous clinical studies have shown that XXMD is an effective alternative therapy not only at the acute stage, but also at the chronic sequelae stage of ischemic stroke. Modern techniques have isolated groups of compounds from XXMD which have shown therapeutic effects, such as dilating blood vessels, inhibiting thrombosis, suppressing oxidative stress, attenuating nitric oxide induced damage, protecting the blood brain barrier and the neurovascular unit. However, which of the active compounds is responsible for its therapeutic effects is still unknown. Emerging studies have screened and tested these active compounds aiming to find individual compounds that can be used as drugs to treat stroke. The present study summarized both clinical evidence of XXMD in managing stroke and experimental evidence on its molecular mechanisms that have been reported recently using advanced techniques. A new perspective has also been discussed with an aim to provide new targets that can be used for screening active compounds from XXMD.

## Introduction

Stroke is the second leading cause of death worldwide, most of which is ischemic strokes (Chang 2015; Katan and Luft, 2018). Stroke accounts for approximately 3–4% of medical expenditure in Western countries (Struijs et al., 2006). Even with dedicated rehabilitation programs, medical costs are estimated to continue to rise, reaching $240.7 billion by 2030 (Ovbiagele et al., 2013). In China, 149 people die from stroke per 100,000 people every year, and the life lost years (YLL) per 100,000 people was 2,633 in 2017, ranked the first in YLL (Zhou et al., 2019).

Currently, the diagnosis of stroke has been standardized with the use of CT, MR and other imaging methods. The course of this disease can be divided into the hyperacute (24 h), acute (1–7 days), subacute (7 days–6 months), and the sequelae stages (6 months later) (Bernhardt et al., 2017), among which the hyperacute and the acute stages are the most important ones that influence the prognosis of patients. The majority of stroke patients remain more or less disabled although various medical treatments are available to restore cerebral blood flow, such as thrombolytic and endovascular therapies. One of the key reasons is that stroke patients are not treated effectively because of delays in arriving at the hospital. With advances in cerebral perfusion imaging and intravascular therapies, the time window for intravenous recombinant tissue-type plasminogen activator (rt-PA) or tenecteplase and intravascular therapies has been extended to 9–24 h (Albers et al., 2018; Nogueira et al., 2018; Ma et al., 2019). However, only a small proportion of patients are selected for these treatments. Even within the 4.5-h time window, only half of patients are able to receive rt-PA thrombolysis. In general, only 30% of patients with acute ischemic stroke received rt-PA (Quain et al., 2008; Meretoja et al., 2012). It is estimated that only 5–13% of patients with hyperacute ischemic stroke are eligible for endovascular therapies within the 6-h time window (Goyal et al., 2016). Although DAWN (Katan and Luft, 2018; Nogueira et al., 2018) and DIFFUSE (Albers et al., 2018) expanded the time window for intravascular therapies with perfusion imaging, the percentage of patients with hyperacute ischemic stroke receiving these therapies remains to be investigated.

For patients who are ineligible for thrombolysis within the time window or arriving at the hospital 24 h after stroke onset, current antiplatelet therapies have shown benefit in reducing mortality and recurrence of stroke. Although fibrin lowering agents and anticoagulants have been proven to be effective, their actual clinical use is limited due to their bleeding risk (Campbell et al., 2019). Other methods, such as conventional supportive treatment which might ameliorate cerebral edema and complications, neuroprotectants like free radical scavengers, therapies that can improve cerebral circulation and nourish nerves, do not have solid evidence to support their use in stroke management. Limb functional position placement, passive limb movement, acupuncture, massage, and other early rehabilitation treatments may be helpful to promote functional recovery of patients with cerebral infarction, but the above-mentioned methods have insufficient evidence and they do not meet the requirements of stroke patients for functional recovery (Hankey 2017).

Ischemic stroke belongs to the category of Wind induced diseases in Traditional Chinese Medicine. XXMD is an effective prescription for the treatment of stroke, which is a breakthrough in the management of stroke in the history of Chinese medicine. It was recorded in “Beiji Qianjin Yaofang Zhufeng” of Qianjin Yaofang (Tang dynasty- Simiao Sun), and later included in “Medical Prescriptions” and “Tangtou Gejue”. Due to its remarkable effect on stroke, it was regarded as “the most important recipe among effective therapies” by many doctors at that time and was prescribed as the first-line wind-dispelling agent, reflecting its importance in the treatment of stroke before Tang and Song Dynasties (Zhu et al., 2002). After Tang and Song dynasties, due to the rise of “non-wind theory” and “internal wind theory,” clinical use of wind dispelling prescriptions was limited. The use of XXMD in the treatment of stroke declined. In the influential Book “General Record of The Holy Universal Relief Zhu Feng,” XXMD was only prescribed for “managing postpartum stroke due to blood deficiency” (a different concept from current stroke) (Chen et al., 2011). During Jin and Yuan dynasties, it was not recommended to treat stroke although its therapeutic effect was never denied. It has survived for thousands of years because of its unparalleled efficacy in stroke management. XXMD is composed of 12 traditional Chinese medicines, including the principal herb [Saposhnikovia divaricata (Turcz. ex Ledeb.) Schischk. [Apiaceae]], 8 minister herbs [Zingiber officinale Roscoe [Zingiberaceae], Ephedra sinica Stapf [Ephedraceae], Paeonia lactiflora Pall. [Paeoniaceae], Conioselinum anthriscoides ‘Chuanxiong’ [Apiaceae], Panax ginseng C.A.Mey. [Araliaceae], Prunus amygdalus Batsch [Rosaceae], Neolitsea cassia (L.) Kosterm. [Lauraceae], Aconitum carmichaeli Debeaux [Ranunculaceae], two assistant herbs (Stephania tetrandra S. Moore [Menispermaceae], Scutellaria baicalensis Georgi [Lamiaceae], and 1 envoy herb (*Glycyrrhiza glabra* L. [Fabaceae]). The composition of XXMD seems strange and difficult for later generations of doctors to understand. In combination with the debate on the etiology of stroke (whether it is due to internal or external wind), many doctors were concerned to use XXMD. Fortunately, this continuous debate has furthered insights into its composition and indications. Recently, clinical application of XXMD has been expanded. The addition and subtraction of certain medicines from XXMD based on the Six Meridians are more consistent with the material basis of TCM therapy for stroke and similar diseases. In this review, clinical studies on XXMD, active compounds of XXMD, and the underlying mechanisms of XXMD will be elaborated, which might point to a new direction for drug development to target stroke patients with residual symptoms and a new perspective was raised with an aim to accelerate the development of new drugs. To find publications on XXMD, 4 English databases (PubMed, EMBASE, Cochrane Central Register of Controlled Trials, and Web of Science), 4 Chinese databases (the Wanfang Database, Chongqing VIP Database, Chinese National Knowledge Infrastructure, and Chinese Biomedical Database), and 2 online clinical trial registration websites (the International Clinical Trials Registry Platform and the Chinese Clinical Trial Registry) were searched up to June 30, 2021. Related trials and systematic reviews were obtained by searching the references of the included studies. Different searching strategies were used, including: “Xiaoxuming Decoction” or “Xiaoxuming Tang” or “Xuming Tang” and “acute cerebral infarction” or “Stroke” or “ischemic attack”. No language restriction was used.

### Clinical Efficacy of XXMD

In the past few decades, modern TCM clinical practice has revealed new indications of XXMD. For this reason, XXMD has been widely used to treat not only stroke but also other diseases. Based on current understanding of the course of ischemic stroke in modern medicine, XXMD has been used to treat ischemic stroke patients at both the acute-subacute and the sequelae stages. Over 320 articles have been published in Chinese, but the majority of them were more like case reports. Therefore, only a few articles that are more or less meeting the criteria of randomized controlled clinical trials were selected to demonstrate the efficacy of XXMD in managing ischemic stroke.

### Acute-Subacute Ischemic Stroke (Within 6 Months of Onset)

A number of studies showed that both the National Institutes of Health Stroke Scale (NIHSS) and the TCM syndrome score of XXMD treated patients were better than those who were treated with western medicine alone. It is proven that XXMD can effectively restore patients’ neurological functions, alleviate clinical symptoms, and accelerate the process of physical recovery.

In clinical practice, XXMD is often used in combination with western medicines or herbal ingredients based on conditions of individual patients. Liu and Qin examined the efficacy of XXMD in combination with alteplase on acute ischemic stroke (6–72 h after stroke onset). It was found that alteplase plus XXMD significantly improved tissue perfusion in the infarct center and the surrounding ischemic area. Meanwhile, it can significantly improve cerebral artery blood supply and hemodynamics, promoting the recovery of neurological functions (Liu and Qin, 2018). In another study, XXMD in combination with dipyridamole significantly decreased the NIHSS of ischemic stroke patients compared with that of ischemic stroke patients in the control group (Chang 2015). Similar findings were reported in a study that applied acupuncture and XXMD to 140 patients with acute ischemic stroke (Ge 2018). Other clinical studies also combined XXMD with Gegen [*Pueraria montana* var. lobata (Willd.) Maesen and S.M.Almeida ex Sanjappa and Predeep] Decoction (Wang and Qian, 2017), or with Huang Qi (*Astragalus mongholicus* Bunge) (Wei and Wang, 2019), or with Xiongdansan Decoction (Li and Cui, 2012) to treat acute ischemic stroke. They all showed that XXMD in combination with other ingredients significantly alleviated symptoms and decreased the NIHSS compared with conservative treatments.

### Sequelae Stage of Ischemic Stroke (>6 Months)

For patients at the sequelae stage of ischemic stroke (>6 months), it was also found that the addition of XXMD to conservative treatments significantly decreased the NIHSS score. Zeng treated 35 patients with stroke sequelae with XXMD and found that it was more superior to conventional western medicines alone in improving neurological functions (Zeng 2018). Hu (2010) also reported that limb functions and cranial CT of patients with ischemic stroke were significantly improved after receiving XXMD. Both Brunnstrom motor functions and Barthel indices of these patients were better than those managed with conservative treatments (Hu 2010; Rui and Huang, 2018).

Ischemic stroke is divided into wind syndrome, heat syndrome, phlegm syndrome, blood stasis syndrome, Qi deficiency syndrome, Yin deficiency and Yang hyperactivity syndrome. XXMD is often used to treat patients with phlegm syndrome, Qi deficiency and blood stasis syndrome. It is especially (Zhao 2012) effective for those with blood stasis syndrome (Sun 1988).

## Components and Active Compounds

XXMD has been proven an effective therapy for ischemic stroke. Development of modern medicine has deepened our understanding of the traditional medicine in many ways. Many researchers have extensively explored pharmacological mechanisms underlying the therapeutic effect of XXMD using emerging chemical and pharmacological techniques. This study aimed to summarize the composition of XXMD, active compounds, pharmacological actions, and pharmacokinetics of these compounds, which provided a comprehensive review of XXMD, an efficacious alternative therapy for ischemic stroke.

## Composition

XXMD, recorded in Beiji Qianjin Yaofang written by Simiao Sun in the Tang Dynasty, is a renowned classical TCM prescription for the treatment of stroke for more than 1,200 years (Zheng and Huang, 2002). In fact, from ancient times to the Song Dynasty, XXMD and its similar prescriptions, such as Xuming Decoction (XMD) and Daxuming Decoction (DXMD), were the first choice of prescriptions for stroke based on the theory of exogenous wind (Zhu et al., 2002). Nowadays, XXMD and similar prescriptions are still widely used to treat stroke in China and other parts of the world. Though slight difference in their compositions of herbs was reported, the main herbs were the same. Therefore, they can be regarded as modified forms of the same prescription. XXMD consists of 12 herbs, including Ephedra sinica Stapf, Saposhnikovia divaricata (Turcz. ex Ledeb.) Schischk, Prunus amygdalus Batsch, Stephania tetrandra S. Moore, Zingiber officinale Roscoe, Panax ginseng C.A.Mey, *Aconitum carmichaeli Debeaux*, *Neolitsea cassia* (L.) Kosterm, *Glycyrrhiza glabra* L, Conioselinum anthriscoides “Chuanxiong,” Paeonia lactiflora Pall, Scutellaria baicalensis Georgi (Table 1). The prescription of XXMD must conform to the basic principle of TCM theory, focusing on replenishing Qi and eliminating pathogenic factors.

**TABLE 1:** Composition of XXMD.

Chinese name	Scientific name	Dosage (g)	Prescription function
Ma huang	Ephedra sinica Stapf	6	Dispel the wind
Fang feng	Saposhnikovia divaricata (Turcz. ex Ledeb.) Schischk	9	Dispel the wind
Sheng jiang	Zingiber officinale Roscoe	10	Dispel the wind
Shao yao	Paeonia lactiflora Pall	9	Replenish the blood
Chuan xiong	Conioselinum anthriscoides ‘Chuanxiong’	9	Replenish the blood
Ren shen	Panax ginseng C.A.Mey	9	Tonify Qi and increase Yang
Xing ren	Prunus amygdalus Batsch	9	Dispel the wind
Gui zhi	Neolitsea cassia (L.) Kosterm	10	Tonify Qi and increase Yang
Fu zi	Aconitum carmichaeli Debeaux	10	Tonify Qi and increase Yang
Fang ji	Stephania tetrandra S.Moore	9	Dispel the wind
Huang qin	Scutellaria baicalensis Georgi	9	Eliminate accumulated heat
Gan cao	*Glycyrrhiza* glabra L	6	Mediate reconciliation between herbs

The characteristics of the formula can be summarized as follows:

1) Wind-dispelling herbs are commonly used, such as Angelica biserrata (R.H.Shan and C.Q.Yuan) C.Q.Yuan and R.H.Shan, Hansenia weberbaueriana (Fedde ex H. Wolff) Pimenov and Kljuykov, Saposhnikovia divaricata (Turcz. ex Ledeb.) Schischk, Stephania tetrandra S. Moore, *Pueraria montana* var. lobata (Willd.) Maesen and S.M.Almeida ex Sanjappa and Predeep, *Gentiana* macrophylla Pall.
2) Warming and cooling herbs are simultaneously used. For example, Zingiber officinale Roscoe, Aconitum carmichaeli Debeaux, and Scutellaria baicalensis Georgi are prescribed together.
3) Tonifying and purging herbs are prescribed together. Saposhnikovia divaricata (Turcz. ex Ledeb.) Schischk and Stephania tetrandra S. Moore [Menispermaceae] dispel the wind, whereas Panax ginseng C.A.Mey, Zingiber officinale Roscoe, Aconitum carmichaeli Debeaux tonify Qi, increase Yang, and replenish the blood.
4) Simultaneous targeting Qi and Blood. Panax ginseng C.A.Mey, Zingiber officinale Roscoe, Aconitum carmichaeli Debeaux can tonify Qi and increase hemopoiesis.

## Active Ingredients or Compounds

Development of modern TCM clinical practice as well as chemical and pharmacological analysis has advanced our understanding of XXMD, which prompts new perspectives to understand XXMD and stroke treatment. Based on the composition of XXMD, functional characteristics of Chinese medicines as well as purified compounds, scientists have systematically studied the active ingredients of XXMD using emerging medical and chemical techniques.

Li et al. (Li et al., 2006a; Li et al., 2007) studied chemical constituents of XXMD dissolved in either ethanol or petroleum ether. Structural analysis of ethanol, water, and petroleum ether extracts using chromatography-mass spectrometry or gas chromatography showed that the water extract was mainly composed of polysaccharides, and no apparent active ingredients were found. In contrast, 16 ethanol-soluble ingredients, such as ephedrine, amygdalin, and N-methylephedrine, were identified from the ethanol extract, and cinnamaldehyde, ethyl palmitate as well as coumarin were isolated and identified from the petroleum ether extract. By taking biological activities of each ingredient, their anti-cerebral ischemia, and anti-senile dementia functions into consideration, it was determined that the active ingredients in the petroleum ether extract of XXMD accounted for about 57% of the total known ingredients (Li et al., 2006b).

In order to accurately understand the material basis of the pharmacological actions of TCM prescriptions, Du (2002) proposed the concept of “effective compound grouping” of TCM prescriptions based on research experience and modern technologies, that is, the organic combination of all pharmacological active ingredients closely related to the clinical indications of TCM prescriptions. This will clarify the material basis and action modes of active compounds to take their clinical effects. Based on this theory, the effective compound grouping of XXMD was studied through comprehensive screening and integrated analysis. Wang et al. reported their preliminary screening result of the effective compound grouping. Petroleum ether, ethanol, and water extracts of XXMD were obtained using the step extraction method. With the assistance of modern separation and high-throughput screening technologies, a group of effective compounds against cerebral ischemia were obtained (Wang et al., 2006). A further study using chemical analysis, pharmacological activity analysis, and comprehensive analysis of activities of multiple targets revealed that 40% of ethanol elution content and the middle layer of the extract showed the strongest activity. Their mixture in a certain proportion comprised the effective compound group against cerebral ischemia (Wang et al., 2011). Using similar methods, Zhang et al. for the first time isolated compounds such as n-octadecanoic acid, n-hexadecanol, baicalin, and baicalein from the effective constituents of XXMD (Zhang et al., 2015). Wang et al. used high performance liquid chromatography-mass spectrometry (HPLC-FTICR-MS and HPLC-LTQMSN) to analyze the chemical constituents of the effective compound group and total extracts of XXMD. A total of 14 ingredients were identified (Wang et al., 2009; Wang et al., 2016). Their further research using HPLC-HRMS combined with mass spectrometry tree similarity filtering (MTSF) technology discovered and identified 68 compounds in the effective group of XXMD (Wang et al., 2014). With the introduction of the concept of effective compound group, it is helpful to comprehensively interpret the theory of Traditional Chinese Medicine and the treatment mode of Traditional Chinese Medicine in scientific languages.

## Pharmacokinetics

Wang et al. (2016) studied the pharmacokinetics of cimicifugin, cimicifugoside, and 5-O-methylvesamitol present in both the effective compound group and the total extract of XXMD. Their results showed that other compounds in the total extract that promoted the absorption of 5-O-methylvisamitol, and promoted the absorption of cimicifugin in the effective compound group. Further study on the pharmacokinetics of effective components in the plasma and the brain showed that levels of flavonoids (including baicalein, oroxyloside, and wogonoside) were high in the plasma, whereas chromones such as cimifugin mainly entered the brain through the blood brain barrier, and alkaloids had higher permeability to the blood brain barrier. Subsequently, it was found that the protective effect of XXMD against cerebral ischemia-reperfusion may be achieved through the synergistic effect of the effective compounds acting on different receptors and signal transduction pathways. In the experimental pharmacodynamics study, Wang found multiple peaks of liquiritigenin, oroxyloside, and wogonoside on the drug concentration-time curves. It was speculated that these three components were transported through the enterohepatic circulation or converted between glycosides and aglycones (Wang 2010). High resolution mass spectrometry confirmed the presence of 247 compounds related to XXMD in rats. These compounds were mainly metabolized by the liver and the kidney through glucuronidation, isomerization, deglycosylation, and subsequently excreted into the bile and the urine (Wang Y. et al., 2012; Wu 2016).

## Mechanisms

The main pathogenic mechanism of ischemic stroke is acute interruption of cerebral blood flow caused by thrombosis, leading to ischemia, hypoxia, degeneration, and necrosis of the local brain tissue. As a result, neurological impairment is presented. The altered state of blood lipid metabolism and hypercoagulability are closely related to the prognosis of patients. Western medicine mainly focuses on symptomatic relief by prescribing anticoagulants, antiplatelets, statins, nutritional support, and early rehabilitation programs, which play an important role in patients’ recovery. However, adverse reactions also occur in some patients, and certain patients do not achieve the expected benefit from these therapies. Studies have shown that XXMD can significantly dilate blood vessels, inhibit thrombosis, improve blood coagulation in blood vessels and regulate lipid metabolism (Table 2).

**TABLE 2:** Summary of the potential mechanisms underlying the therapeutic effect of XXMD on stroke.

Regulate blood flow status	Dilate blood vessels
	inhibit thrombosis
	regulate blood coagulation status
	regulate blood lipids
brain protection	anti-oxidative stress
	anti-NO damage
	anti-neuroinflammation
	protect the neurovascular unit
	protect the blood brain barrier

Vasodilation: insufficient blood supply caused by cerebral vasoconstriction is one of the pathophysiological mechanisms of ischemic stroke. Vasodilating drugs can restore blood supply and subsequent energy supply to ischemic regions, which provides material basis for subsequent anti-ischemia-reperfusion injury therapies and recovery of neurological functions. Studies have shown that XXMD can significantly improve cerebrovascular blood supply in animals with cerebral ischemia and cerebral hemorrhage, reduce the permeability of capillaries in brain tissues, and alleviate the pathological changes of the blood brain barrier (Chen and Wu 1991; Chen et al., 1997; Ye et al., 1999). Lu et al. (2018) constructed a network of “composition-vasomotor G protein-coupled receptor (GPCR) targets” for XXMD, and identified five types of GPCRs related to vasodilation and constriction: serotonin 1A and 1B receptors (5-HT1AR, 5-HT1BR), angiotensin II type 1 receptor (AT1R), β2 adrenergic receptor (β2-AR), urotensin II receptor (hUTR), the endothelin receptor B (ETB). It was found that the majority of the chemical compounds of XXMD acted on different vasomotor related GPCR targets, and only a small number of compounds could simultaneously act on multiple GPCR targets to take synergistic vasodilating effects.

Inhibition of thrombosis and improvement of intravascular coagulation: it has been reported that intragastric infusion of XXMD for 5 days prolonged the coagulation time of mice (Lan et al., 2013a) and inhibited the thrombus formation in rats (Chen and Wu, 1991). *In vitro* administration of XXMD significantly suppressed the maximum platelet aggregation rate induced by ADP and prolonged the maximum platelet aggregation time by 50%. Interestingly, intravenous administration of XXMD significantly increased the blood flow of the internal carotid artery in dogs, reduced the cerebrovascular resistance, slightly accelerated the heart rate, without apparent influence on the blood pressure. In addition, studies found that the NIHSS, levels of fibrinogen and D-dimers were significantly lower in the group managed with conventional treatments plus XXMD than in the control group managed with conventional treatments alone, indicating that XXMD has a significant therapeutic effect on acute cerebral infarction. It can effectively suppress coagulation and improve neurological functions (Fan et al., 2018; Li and Qin 2018; Liu and Zeng 2018; Shen 2018).

Regulation of blood lipids: Guan and Wang compared changes of the body weight, total cholesterol, and triglyceride before and after 30 days of intragastric administration of XXMD to quails which were modeled by feeding high-fat diet. It was found that XXMD at a low-dose (9 g/kgd) significantly decreased levels of the total cholesterol and triglycerides observed in the high-fat group (Guan and Wang, 1996). It is known that high levels of cholesterol and triglycerides are associated with atherosclerosis which is one of the predominant mechanism leading to brain ischemia.

Anti-cell apoptosis: He et al. confirmed that the effective compounds of XXMD could mitigate neuronal apoptosis by increasing the ratio of B-cell lymphoma-2 (Bcl-2)/Bax-apoptosis related genes in the brain and inhibiting the downstream cascade of caspase induced apoptosis (He et al., 2012). Zhu et al. simulated the ischemia-reperfusion injury both *in vivo* and *in vitro*. The therapeutic effect of XXMD was evaluated by assessing neural response to ischemic injury, the activity of caspase-3 in the hippocampal CA1 region, and the expression of Bcl-2. Cognitive performance was assessed using the Morris water maze test 7 days after the ischemic injury. It was found that XXMD increased the density of viable neurons in the hippocampal CA1 region, attenuated spatial cognitive deficits, inhibited the activation of caspase-3, and up-regulated the expression of Bcl-2. These indicate that anti-apoptosis is one of the mechanisms underlying the therapeutic effect of XXMD (Zhu et al., 2010).

Anti-oxidative stress: it is well known that blood flow is interrupted after ischemic stroke onset, and reperfusion to the brain tissue triggers a series of mechanisms, such as accumulation of large amounts of calcium ions, massive production of oxygen free radicals, and acute cell edema, which leads to necrosis of neural cells and damage of the neurovascular unit. Thrombolytic therapies or intravascular recanalization rapidly increased the blood flow to the brain tissue, which potentially aggravated the injury of the ischemic tissue and brain edema. Consequently, brain tissue damage became more severe, which is an important factor contributing to the deterioration of ischemic stroke patients. However, for the majority of ischemic stroke patients, recanalization therapies generally improve the neurological functions of these patients. Timing of starting these therapies must be an important determinant of the outcome of these patients. The balance between increased blood supply to the microcirculation along with the subsequent improvement of neuronal metabolism and ischemia-reperfusion induced injury remains to be investigated.

Animal experiments showed that the effective ingredients of XXMD at both low and medium doses could inhibit lipid peroxidation, increase the activity of glutathione-peroxidase (GSH-Px), leading to suppression of oxidative stress response. Cheng et al. found that the addition of XXMD to conventional treatments significantly restored neurological functions as shown by the decreased NIHSS and the increased BI score. This was mainly due to suppression of levels of superoxide dismutase and chemokine MCP-1, increased levels of BDNF, and increased blood supply to the brain as well as improved hemodynamics (Cheng et al., 2019). In addition, XXMD can significantly reduce the expression of inflammatory factors like IL-6 (IL-6), tumor necrosis factor -α (TNF-α), and hypersensitive C-reactive protein (hs-CRP) in patients with acute ischemic stroke (Xie et al., 2019).

Anti-nitric oxide (NO) induced damage: nitric oxide is a highly active and diffusible molecule with a very short half-life. NOS is present in endothelial cells, neural cells, nerve fibers, and glial cells, and it has a variety of physiological functions in organisms, such as dilating blood vessels, lowering the blood pressure, suppressing platelet aggregation and adhesion. When cerebral ischemia occurs, NO has a dual effect. For example, experimental data have shown that NO exerts a protective effect at the early stage of ischemia and a neurotoxic effect at the late stage. Improving microcirculation in the ischemic penumbra salvages the dying neurons, whereas necrosis in the infarct core aggravates tissue damage. Wang and Tang found that the level of plasma NO and the activity of NOS in stroke model rats were significantly decreased after intragastric administration of XXMD, suggesting that the reduction of plasma NO content and NOS activity in rats with acute ischemic stroke might be one of the mechanisms of XXMD to ameliorate cerebral ischemia injury (Wang and Tang, 2009). It has been reported that XXMD may play a protective role in focal cerebral ischemia by regulating the oxidation-antioxidation balance in the brain and by decreasing the activity of inducible nitric oxide synthetase (iNOS) (Wang YH. et al., 2012).

Anti-neuroinflammation: it is clear that cerebral infarct activates microglia and astrocytes to engulf the necrotic tissue. This consequently results in the development of fibrotic scars. This neuroinflammatory response is further aggravated by cytokines released by microglia and astrocytes (Shi et al., 2019; Liu et al., 2020). Therefore, a vicious cycle is established. In addition, massive production of reactive oxygen species and peroxynitrite leads to the enlargement of ischemic lesions. Clinical studies have also shown that peripheral systemic inflammatory response index (SIRI) is negatively correlated with the outcome of ischemic stroke patients undergoing endovascular thrombectomy though complete recanalization has been achieved in these patients (Lattanzi et al., 2021). The neutrophil/lymphocyte ratio was also correlated with symptomatic hemorrhagic transformation in acute ischemic stroke patients who completed revascularization (Świtońska et al., 2020). XXMD significantly reduced the expression of inflammatory cytokines in patients with acute ischemic stroke as stated above (Xie et al., 2019). Furthermore, XXMD decreased the expression of TLR4 and MyD88 in microglia in both *in vitro* and *in vivo* experiments (Cheng et al., 2019; Xiao et al., 2019). A network pharmacology study reported that multiple signal transduction pathways, such as IL-17, TNF, AGE-RAGE, were regulated by XXMD (Wu et al., 2021). In hemorrhagic stroke, increased matrix metalloproteinases (MMPs) play an important role in the neuroinflammatory response not only through degrading proteins in the extracellular matrix and tight junction proteins but also through activating a number of inflammatory mediators (Lattanzi et al., 2020). This was also observed in ischemic stroke models. Treatment with XXMD significantly downregulated the level of MMP9 in the ischemic stroke rat model (Lan 2014). These suggest that XXMD can suppress neuroinflammation in both ischemic and hemorrhagic stroke through multiple pathways and restore neurological functions of both patients and stroke models.

Protection of the neurovascular unit: in an experimental study, Wang et al. found that the effective compounds of XXMD alleviated the reduction of cerebrovascular reserve and the reduction of the number of neurons as well as the altered morphology due to chronic ischemia in rats. It also effectively decreased the activation of astrocytes (Wang Y. et al., 2012). The other study suggested that the protective effect of XXMD on the neurovascular unit may be the result of activation of the phosphatidylinositol-3 kinase/Akt pathway. In other studies, though the concept of neurovascular unit was not mentioned, it is plausible to draw a conclusion that XXMD protects the neurovascular unit through its anti-apoptosis, anti-oxidative stress, anti-neuroinflammatory and other mechanisms.

Protection of the blood-brain barrier: intragastric infusion of different doses of XXMD to the ischemia-reperfusion stroke model rats resulted in not only reduced size of cerebral infarcts, improved behavioral performance, but also decreased cell ultrastructural damage, and decreased blood-cerebrospinal fluid barrier permeability (Yang et al., 2021). It might be the consequence of decreased expression of matrix metalloproteinase-9, -2, and vascular endothelial growth factor (Lan 2014). Zhou et al. found that XXMD attenuated the loss of tight junction related protein zona occludens-1 (ZO-1), maintained the structural integrity of the tight junction complex, inhibited the excessive release and expression of aquaporin 4 (AQP4) mRNA, and ameliorated cerebral ischemia and edema in rats with focal cerebral ischemic stroke (Zhou et al., 2014). (Lan et al., 2013b). found that XXMD reduced mitochondrial autophagy by down-regulating expression levels of autophagy genes LC3, Beclin1, Lamp1, and mitochondrial P62, playing a neuroprotective role (Lan et al., 2018).

In conclusion, XXMD is an effective alternative therapy for ischemic stroke through regulating the blood flow of patients and protecting the brain tissue. It can dilate blood vessels, reduce thrombosis, regulate blood coagulation and blood lipids to improve the blood flow status of patients, exerting the protective effect. On the other hand, XXMD can not only inhibit the oxidative reaction, calcium overload, inflammatory reactions secondary to neuronal injury, protect the neurovascular unit, but also reduce brain edema and protect the blood-brain barrier.

## Perspectives

XXMD has a long history and modern clinical evidence in the treatment of ischemic stroke, but the results of multi-center randomized clinical trials are still lacking. Modern biochemical methods have deepened the understanding of the composition and effective compound groups of this traditional recipe, but the individual effective ingredients have not been confirmed.

Although the mechanism of XXMD in managing ischemic stroke has been widely explored, there are still many challenges. Studies in recent years have shown that microvascular pericytes will contract after ischemia (Nikolakopoulou et al., 2019), and erythrocytes and leukocytes will accumulate in the microcirculation, leading to microcirculation ischemia. Even after recanalization of large vessels, microcirculation remains occluded (Hu et al., 2017). Improvement of microcirculation by dilating pericytes might be a future research direction for stroke patients with residual symptoms (Cheng et al., 2018). At present, research on the target of Traditional Chinese Medicine is still focused on the oxidative stress reaction due to ischemia-reperfusion injury. How to scavenge free radicals is the main aim of research, and the role of pericytes in this process may be neglected. Therefore, the regulation of pericyte dilation and contraction may be an important target for future research. It is expected to find effective drugs for ischemic stroke patients with residual symptoms using this target.

As mentioned above, XXMD is not only effective for ischemic stroke, but also effective for hemorrhagic stroke (Chen and Wu 1991; Chen et al., 1997; Ye et al., 1999). This raises the hypothesis that both ischemic and hemorrhagic stroke must share certain pathogenic mechanisms. Disentangling these common mechanisms might lead to the application of XXMD to hemorrhagic stroke patients. This will solve the dilemma in managing hemorrhagic stroke patients due to the lack of effective medications.
